# Effectiveness of Non-Pharmacological Interventions for Older Adults with Dementia: An Umbrella Review

**DOI:** 10.1093/geroni/igaf122.3921

**Published:** 2025-12-31

**Authors:** Taeko Saito, Natsumi Shimizu, Li Yao

**Affiliations:** Nippon Medical School, Inzai, Chiba, Japan; Musashino University, Tokyo, Tokyo, Japan; Chiba University, Chiba, Chiba, Japan

## Abstract

Dementia is a major global public health concern, with older adults with dementia particularly vulnerable to complications such as delirium and psychological symptoms of dementia (BPSD). Non-pharmacological interventions (NPIs) have been found to be effective for these symptoms, and several meta-analyses have shown NPIs are effective in reducing BPSD delirium. However, evidence on NPIs remains fragmented across existing reviews and there has been no comprehensive evaluation of the strength and validity of NPIs. Therefore, this study aimed to identify assess the previous evidence using umbrella review consolidates data from systematic reviews and meta-analyses. A comprehensive literature search was conducted across PubMed, Medline, Web of Science, CHINALE, PsycINFO from inception to March 2025, 15systematic reviews that met the inclusion criteria were identified. Two reviewers independently screened selected studies, extracted data, and assessed methodological quality using the AMSTAR 2 tool. NPIs included interventions using ICT, exercise, music, light therapy, and various combinations of these approaches. In addition, BPSD and delirium symptoms such as agitation, depression, anxiety, and cognitive function were also addressed. A meta-analysis of non-pharmacological interventions (NPI) for these symptoms was conducted. As a result, NPI was suggested to be effective for several symptoms. Providing integrated knowledge on the current initiatives, this umbrella review study is the first step towards better crafted interventions that support NPI to elderly people with dementia and designing appropriate interventions for BPSD and delirium.

